# Surface cooling for induction of mild hypothermia in conscious healthy volunteers - a feasibility trial

**DOI:** 10.1186/cc10506

**Published:** 2011-10-22

**Authors:** Christoph Testori, Fritz Sterz, Wilhelm Behringer, Alexander Spiel, Christa Firbas, Bernd Jilma

**Affiliations:** 1 Department of Emergency Medicine, Medical University of Vienna, Währinger Gürtel 18-20, 1090 Vienna, Austria; 2 Department of Clinical Pharmacology, Medical University of Vienna, Währinger Gürtel 18-20, 1090 Vienna, Austria

**Keywords:** human experimentation, hypothermia, magnesium, myocardial infarction, safety, stroke, temperature

## Abstract

**Introduction:**

Animal and human studies suggest beneficial outcome effects of mild hypothermia for stroke, for acute myocardial infarction, and for cardiogenic shock. The aim of this study was to investigate the feasibility and safety of non-invasive surface cooling for induction and maintenance of mild hypothermia (32 to 34°C) in healthy, conscious volunteers.

**Methods:**

The trial was set at a clinical research ward in a tertiary care center, and included 16 healthy male volunteers 18 to 70 years old. Surface cooling was established by a novel non-invasive cooling pad with an esophageal target temperature of 32 to 34°C and maintenance for six hours. Shivering-control was achieved with meperidine and buspirone and additional administration of magnesium in eight subjects.

**Results:**

The primary endpoint to reach a target temperature of 32 to 34°C was only reached in 6 of the 16 participating subjects. Temperatures below 35°C were reached after a median cooling time of 53 minutes (38 to 102 minutes). Cooling rate was 1.1°C/h (0.7 to 1.8°C). Additional administration of magnesium had no influence on cooling rate. At no time during the cooling procedure did the participants report uncomfortable conditions for which termination of cooling had to be considered. No severe skin damage was reported.

**Conclusions:**

Cooling to body temperature below 35°C by the use of non-invasive surface cooling is feasible and safe in conscious healthy volunteers. Further studies are needed to investigate an altered cooling protocol to achieve temperatures below 35°C.

**Trial Registration:**

ISRCTN: ISRCTN50530495

## Introduction

Therapeutic hypothermia improves neurological outcome and reduces the risk of death in patients after cardiac arrest [1,2]. In recent years, growing evidence in animal and human studies have documented or suggested the beneficial outcome effects of mild hypothermia also for stroke [3-11], for acute myocardial infarction [12-16], and for cardiogenic shock [17]. Rather than interacting on a particular pathway of post-ischemic reperfusion damage hypothermia influences multiple reactions leading to cell death [18-20].

So far, the majority of therapeutic hypothermia research has involved cardiac arrest patients, in whom the induction and maintenance of mild hypothermia is facilitated by post-anoxic coma, anesthesia and paralysis to avoid shivering. The typical patient presenting to an emergency department with ischemic stroke or acute myocardial infarction is awake and does not present in a clinical status that necessitates intubation and neuromuscular blockade. The induction of mild hypothermia in these patients remains a challenge. Recent studies investigated various methods for inducing mild hypothermia in awake volunteers or awake patients, such as infusion of cold saline [21,22], surface cooling with circulating chilled water through energy transfer pads placed on the patients' skin [14,23,24], or endovascular cooling catheters [4,15,25-28]. However, infusion of cold saline does not provide sustained mild hypothermia [29], and cooling devices are bulky and need an electrical power supply, thus preventing the use of these devices in the out-of-hospital setting. Endovascular cooling necessitates the puncture of a main venous blood vessel with all its complications, such as arterial puncture, hematoma, pneumothorax, venous thrombosis and catheter-related infections [30-33]. Recently, a simple to use, non invasive cooling pad, independent of an energy source during use, was developed, which proved to rapidly induce mild hypothermia in patients after cardiac arrest in the out-of-hospital setting [34] and in the emergency department [35].

The aim of this study was to investigate the feasibility and safety of non-invasive surface cooling for induction and maintenance of mild hypothermia (32 to 34°C) in healthy, conscious volunteers.

## Materials and methods

### Study design

This study was conducted according to the principles of the Declaration of Helsinki (Version 4, 2004) and was approved by the Ethics Committee of the Medical University of Vienna. This was a prospective interventional study in a cohort of 16 healthy volunteers. Subjects were informed verbally and in writing about the aims of the study. Each volunteer provided written informed consent. Financial compensation was according to standard operating procedures of the Department for Clinical Pharmacology, Medical University of Vienna.

### Study setting and population

Volunteers 18 to 70 years old were included. We excluded pregnant women, subjects with a known pre-existing cardiopulmonary disease or pre-existing malignancy, those with pre-existing coagulopathy, with an active dermatologic condition, with a current treatment with monoamine oxidase inhibitors, with previous or current drug abuse, and with a known allergy to the study medication. Core temperature had to be below 37°C at the start of the cooling procedure.

### Study protocol

Prior to enrolment, a general health check was performed for each subject. This examination included a general physical examination, lab tests, resting ECG and chest X-ray. Abstinence from alcohol, tobacco and strenuous exercise for 12 hours before the study were requested. Subjects were asked to only consume a light meal before their study day at the research ward to minimize any potential nausea caused by meperidine infusion. Compliance with these requests was confirmed by interview before each trial. Before initiation of cooling, volunteers were asked about any symptoms of infection to avoid aggravation of an incipient disease.

*Medication: *After baseline measurements, meperidine (Alodan^®^, Gerot, Vienna, Austria) 1 mg/kg bolus, followed by 30 mg/h intravenously, and on oral dose of buspirone (Buspar^®^, Bristol-Myers Squibb, Meymac, France) 30 mg was given to prevent shivering. A single bolus of granisetron hydrochloride (Kytril^®^, Roche Austria, Vienna, Austria) 3 mg was administered intravenously to prevent nausea. A continuous fluid drip with an isotonic electrolyte solution (Elomel iso^®^, Fresenius Kabi Austria, Graz, Austria) with an infusion rate of 100 ml/h was administered throughout the cooling period. Because of problems reaching the target temperature of 32 to 34°C we decided to additionally administer a bolus of 4 g magnesium sulfate (MgSO_4_) over 30 minutes followed by a continuous intravenous drip of 2 g/h for 150 minutes. As such, the administration of magnesium was limited to eight volunteers.

*Cooling procedure: *The cooling pads (EMCOOLSpad^®^, Emcools AG, Pfaffstaetten, Austria), each 20 × 30 cm, consist of multiple cooling cells filled with a patented cooling gel. The inner layer is a biocompatible film that adheres to the patient's skin on application and provides intimate pad to skin contact for efficient heat transfer. The cooling units were stored in a cooling box at -2°C before use. Six cooling units were applied on the back, thorax, abdomen and thighs of the volunteers. In previous studies in patients after cardiac arrest, a temperature drop was observed after the removal of the cooling units [34,35]; thus, in this study, the cooling units were removed when a core temperature of 35°C was reached. Target core temperature was 32 to 34°C and maintained for six hours. The following algorithm was applied to maintain the temperature: if the temperature did not drop below 34.3°C, or the temperature started to increase before reaching 34.3°C, two cooling units were applied on the thorax and abdomen, until the temperature reached 34°C, then the cooling units were removed (cooling units were exchanged when thawed). If the temperature dropped below 33°C, a warming blanket was applied until the temperature reached 33.1°C.

After six hours, the volunteers were covered with a blanket and allowed to re-warm. At a temperature of 35°C, meperidine was stopped, and the volunteers were observed for additional 16 hours.

### Measures

Core temperature was measured continuously with a temperature probe (Mon-a-therm^® ^9Fr/Ch; Tyco Healthcare, Mansfield, MA, USA) advanced into the esophagus, and recorded continuously. The esophageal probe was inserted through the nose. To avoid discomfort and vomiting, local anesthesia with lidocain spray 2% (Xylocain 2%^®^, AstraZeneca GmbH, Wedel, Germany) was applied. Blood pressure was measured non-invasively with Riva-Rocci (Philips Healthcare; Andover, MA, USA), and recorded every 15 minutes. ECG, heart rate and peripheral oxygen saturation were recorded continuously. An intravenous line was placed into a peripheral vein for administration of medication and for blood sampling. A comfort score was recorded every 10 minutes during initial cooling, and every 30 minutes during maintenance cooling. The comfort was measured by a 5-point scale: 1 = extremely uncomfortable, immediate withdrawal; 2 = very uncomfortable, withdrawal imminent; 3 = unpleasant, no reason for withdrawal at this time; 4 = no specific sensations, continue the observation; 5 = pleasant sensation, continue the observation. Volunteers were monitored for shivering using a 4-point scale [36]: 0 = no shivering evident; 1 = isolated facial or masticatory fasciculation; 2 = peripheral shivering; 3 = uncontrolled rigor. In case of shivering or a comfort score of ≤ 3, a meperidine 20 mg bolus, followed by an increase of the meperidine drip rate by 5 mg/h, was given. At the end of the observational period, the skin of all subjects was examined by a dermatologist using a 5-point scale (severe - frost bite; moderate - skin trauma; medium - red skin; mild - pink skin; no visible skin irritation). Due to feasibility and the convenience of the volunteers, the period of active cooling was set at six hours.

### Data analysis

Continuous variables are given as mean ± standard deviation, or as median and the minimum/maximum range, if not normally distributed. Nominal data are given as counts and percentage of total number. For group comparisons of continuous variables the Student's *t*-test, or the Mann-Whitney-U-test were used. For group comparisons of proportions, the Chi Square test or Fishers Exact test were used. For comparisons within a group, the paired t-test or Wilcoxon Signed Rank test were used. Cooling rates were calculated by the time needed from baseline temperature to an esophageal temperature of 35.0°C. SPSS software (version 16.0, SPSS Inc., Chicago, IL, USA) and Microsoft Excel (version 12.0 for Mac, Microsoft Corp., Redmond, WA, USA) were used for statistical analysis.

## Results

All 16 healthy male volunteers aged between 21 and 47 years followed the protocol. Median age was 32 years (21 to 47 years), and median body mass index was 23.3 kg/m^2 ^(19.5 to 29.1 kg/m^2^); four volunteers were smokers.

**Figure 1:**
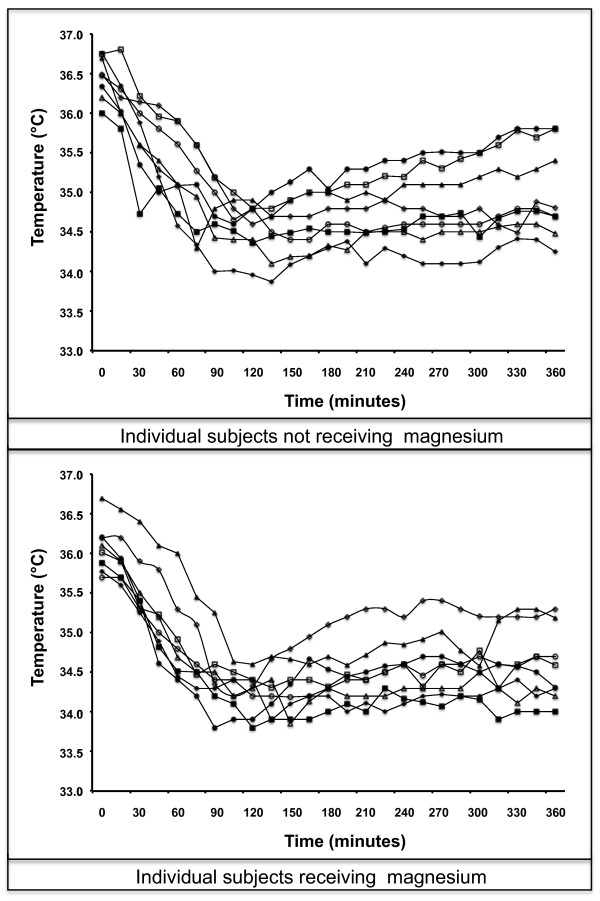
**Temperature curves of all subjects**. Upper figure: subjects not receiving MgSO_4_; lower figure: subjects receiving MgSO_4._

In 11 volunteers, all cooling units had to be exchanged before reaching an esophageal temperature of 35°C, because they were completely thawed. Esophageal temperature decreased from baseline median 36.2°C (35.7 to 36.8°C) to 35°C within a median time of 53 minutes (38 to 102 minutes), which translates into a median cooling rate of 1.1°C/h (0.7 to 1.8°C/h). We found no correlation between body mass index and cooling rate (*P *= 0.62). Only in one volunteer, core temperature dropped below 34°C after removal of the six initial cooling units. All volunteers started to rewarm after initial cooling so that two maintenance-cooling units had to be applied and had to be replaced four times (two to five times) because they were completely thawed. Esophageal temperature could be decreased to a minimum temperature of 34.4°C (33.7 to 34.7°C) during the cooling procedure. An esophageal temperature below 34°C could be established in six subjects. After six hours of cooling, esophageal temperature was 34.7°C (34.0 to 35.8°C). (Figure 1)

There was no statistically significant difference in the time to achieve 35°C between those volunteers receiving MgSO_4 _and those not receiving MgSO_4 _(48 minutes (38 to 93 minutes) vs. 67 minutes (44 to 102 minutes); *P *= 0.13)). Minimal temperature reached was significantly lower in volunteers receiving MgSO_4 _(34.2°C (33.7 to 34.4°C) vs. 34.4°C (33.9 to 34.7°C); *P *= 0.03)). Temperatures at the end of the cooling period were lower in subjects receiving MgSO_4 _(34.5°C (34.0 to 35.3°C)) as compared to those not receiving MgSO_4 _(34.8°C (34.3 to 35.8°C)), although this was not statistically significant (*P *= 0.09). Serum magnesium levels significantly increased after infusion of the bolus from median 0.87 mmol/l (0.82 to 0.97 mmol/l) to 1.38 mmol/l (1.12 to 1.51 mmol/l; *P *< 0.01). Plasma concentrations further increased to a level of 1.54 mmol/l (1.38 to 1.59 mmol/l) at the end of infusion time of 150 minutes and a cumulative dosage of 9 g MgSO4. Magnesium concentrations prior to discharge (0.90 mmol/l; 0.85 to 0.98 mmol/l) were not significantly different from baseline (*P *= 0.45).

Heart rate, respiratory rate, oxygen saturation and systolic and diastolic blood pressure decreased significantly during cooling (Table 1), but none of the volunteers complained about dizziness or any other signs of hemodynamic impairment. There were no significant differences in the vital signs during the cooling period between volunteers receiving MgSO_4 _and volunteers not receiving MgSO_4_, except systolic blood pressure, which was significantly lower in volunteers receiving MgSO_4 _(113 mmHg (96 to 121 mmHg) vs. 122 mmHg (114 to 133 mmHg); *P *= 0.015)).

**Table 1:** Vital signs

	Baseline	**Cooling**^**a**^	*P*-value
Heart rate, minute^-1^	70	49	< 0.01
MMR^b^	55 to 96	43 to 101	
Peripheral oxygen saturation, %	98	95	< 0.01
MMR^b^	93 to 100	90 to 98	
Respiratory rate, minute-1	16	10	< 0.01
MMR^b^	10 to 21	7 to 13	
Systolic blood pressure, mmHg	134	117	< 0.01
MMR^b^	113 to 149	96 to 133	
Diastolic blood pressure, mmHg	68	58	< 0.01
MMR^b^	54 to 94	37 to 67	

^a^lowest value during cooling procedure

^b^Minimum-maximum range

During the procedure participants never reported uncomfortable conditions necessitating termination of cooling. There was no difference in comfort scale between groups receiving MgSO4 or not. Shivering was observed in four volunteers (two receiving MgSO4), which could be easily controlled by the adjustment of meperidine drip. Cumulative meperidine dose was 5.8 mg/kg (4.1 to 11.5 mg/kg) with no difference in patients additionally receiving MgSO_4 _and those not receiving MgSO4 (*P *= 0.75).

When removing the cooling pads after initial cooling to a core temperature of 35°C, red skin was observed in all 16 subjects. After re-warming to temperatures > 36°C, the skin irritation was no longer evident in two subjects, changed to mild skin irritations (pink skin) in 13 subjects, and remained as red skin in one subject. Prior to dismissal from the study lab, 10 volunteers still had pink skin. All subjects were followed up for seven days. Skin irritation decreased in all subjects during the follow-up period.

## Discussion

This trial showed that cooling by a novel non-invasive surface cooling pad to temperatures < 35°C is feasible and safe in healthy volunteers. Although the primary endpoint to reach a target temperature of 32 to 34°C was only reached in 6 of the 16 participating subjects. With the presented study protocol we were not able to achieve predefined ranges of mild hypothermia of 32 to 34°C.

Most of these patients with acute stroke or acute myocardial infarction are awake and do not present in a clinical status that necessitates intubation and neuromuscular blockade. Furthermore, patients with an acute stroke require serial neurological assessments making deep sedation or anesthesia a contraindication. Meperidine and buspirone were chosen because of their previously described effects on thermoregulation in human beings. The efficacy and use of meperidine and buspirone to suppress shivering have been well described in the anesthesia literature and previous cooling studies [21]. MgSO_4 _did not markedly improve the cooling rate or comfort of subjects in our study, but might aid to achieve and maintain lower temperatures for a longer time period. In contrast to our results, Zweifler *et al*. showed that the additional administration of MgSO_4 _improved the comfort in surface cooling of mildly sedated spontaneously breathing subjects [37]. Zweifler used a bolus infusion of 4 to 6 g MgSO_4_, followed by a continuous drip of 1 to 3 g per hour. Taking into account that Zweifer *et al*. administered MgSO_4 _over six hours, the cumulative MgSO_4 _dose was higher in their study. The infusion rate during the first 180 minutes was similar to ours, and so their conclusion is not supported by our data. In our study, a bolus infusion of 4 g MgSO_4_, followed by a continuous-drip of 2 g per hour for 150 minutes significantly reduced systolic blood pressure in our subjects. This finding might be important for future treatment with hypothermia in patients with myocardial infarction and cardiogenic shock, or patients with stroke and hypotension, in whom a further drop in blood pressure will be detrimental.

In a former study in cardiac arrest patients with the same cooling method, cooling was more effective in terms of cooling rate and lowest temperature achieved. [34] This might be explained by the use of only six pads for initial cooling in our study as compared to 10 cooling units used in cardiac arrest patients [34]. In the current study, pads were stored at approximately -2°C as compared with a storage temperature of -9°C in cardiac arrest patients [34]. In addition, awake subjects might have a more evident counter-regulatory response against cooling efforts, as compared with sedated and paralyzed patients after cardiac arrest.

The infusion of cold saline proved to be a simple method to induce mild therapeutic hypothermia in cardiac arrest patients, but needs an additional cooling method to maintain mild hypothermia [15,38,39]. However, in awake subjects, cold saline alone might be ineffective to induce mild hypothermia [22]. In addition, the volume load of 30 mL/kg to induce mild hypothermia might be harmful in patients with myocardial infarction and left ventricular dysfunction.

Our study has several limitations worth mentioning. The trial was conducted as a feasibility trial in healthy volunteers and may or may not be extrapolated to patients with acute stroke or acute myocardial infarction. Thermoregulation might be different in elderly or sick patients. The major limitation of the study is the fact that the target temperature of 32 to 34°C was not achieved in two-thirds of the subjects. This might be due to the removal of the cooling pads at 35°C, based on our expectation of a more evident drop in temperature after removal of the cooling units as described in cardiac arrest patients [34]. As a consequence, maintenance cooling in the predefined ranges was not achieved. However, in a preliminary study in patients with myocardial infarction, lowering the core temperature to levels of only 35°C showed a significant reduction of infarct size. Animal models suggest a more distinctive effect of hypothermia in myocardial infarction if temperature management is initiated early in the pre-reperfusion period [40]. Recently, Götberg *et al*. stated that in myocardial infarction, hypothermia should be initiated as soon as possible without delaying reperfusion [41]. Given that this cooling method can be initiated by paramedics in the field, and a core temperature of 35°C was reached within 53 minutes in the current study, patients with myocardial infarction might be cooled to therapeutic levels of hypothermia already during transport to the hospital.

## Conclusions

Cooling to a body temperature below 35°C by the use of non-invasive surface cooling is feasible and safe in conscious, healthy, mildly sedated volunteers. Further studies are needed to investigate: 1) an altered cooling protocol to achieve temperatures below 35°C, and 2) the feasibility and safety of surface cooling with these pads in awake patients with stroke or acute myocardial infarction.

## Key messages

• Surface cooling in healthy, mildly sedated volunteers is feasable and safe.

• An altered cooling protocol is required to achieve lower temperatures. The additional administration of magnesium does not lead to faster cooling rates.

## Abbreviations

MgSO_4_: magnesium sulfate

## Competing interests

CT, CF, BJ and AS have no competing interests. FS holds a patent relating to the content of the manuscript, but has never received reimbursements, fees, funding or a salary from an organization or company relating to the content of the manuscript. WB is a co-founder, shareholder and paid medical advisor of EMCOOLS (Emergency Medical Cooling Systems AG). EMCOOLS provided the cooling device, but was not involved in creating the design, data management and data analysis of the study, or the manuscript preparation or authorship.

## Authors' contributions

CT contributed to the conception and design of the study, the acquisition of the data, the analysis and interpretation of the data, and drafting of the manuscript. FS contributed to the conception and design of the study, the acquisition of the data, the analysis and interpretation of the data and the revision of the manuscript. WB contributed to conception and design of the study and revision of the manuscript. AS and CF contributed to the acquisition of the data and revision of the manuscript. BJ contributed to the conception and design of the study, the acquisition of the data, the analysis and interpretation of the data, and the revision of the manuscript. All authors read and approved the final manuscript
